# Framework for assessing the performance of the Ghanaian health system: study protocol

**DOI:** 10.1186/s12961-021-00802-1

**Published:** 2021-12-20

**Authors:** Emmanuel Kumah, Samuel E. Ankomah, Adam Fusheini, Peter Agyei-Baffour, Martin A. Ayanore, Richard K. Asravor, Felix O. Kesse, Emmanuel Mwini, Godfred Otchere

**Affiliations:** 1 Department of Health Administration and Education, Faculty of Science Education, University of Education, Winneba, Ghana; 2 Department of Preventive and Social Medicine, Dunedin School of Medicine, University of Otago, Dunedin, New Zealand; 3 Center for Health Literacy and Rural Health Promotion, P.O. Box GP1563, Accra, Ghana; 4 Department of Health Policy, Management and Economics, School of Public Health, Kwame Nkrumah University of Science and Technology, Kumasi, Ghana; 5 Department of Health Policy, Planning and Management, School of Public Health, University of Health and Allied Sciences, Ho, Ghana; 6 Ghana Technology University College, Accra, Ghana; 7 Department of Administration, Kwesimintsim Government Hospital, Takoradi, Western Region Ghana; 8 Planning, Monitoring and Evaluation Directorate, Ministry of Health, Accra, Ghana; 9 Faculty of Humanities, Center for Medicine and Society, University of Freiburg, Freiburg, Germany

**Keywords:** Health systems, Performance assessment framework, Health indicators, Developing country, Health system in Ghana

## Abstract

**Background:**

Assessing the performance of health systems through quantitative and qualitative methods is recognized as an effective approach to strengthening national health systems. However, while many high-income countries have institutionalized health system performance assessment (HSPA) as an integral component of their respective health systems, few studies on HSPA have been documented in low- and middle-income countries, including Ghana. This study aims at providing a comprehensive framework for periodic assessment of the performance of the entire health system in Ghana.

**Methods:**

The study will have four work packages. First, a structured review will be conducted to identify both international and national HSPA frameworks that could be applied to the Ghanaian context. Second, based on the structured review, an assessment framework tailored to the Ghanaian health system context will be developed. Third, the draft framework will be presented and discussed with experts and stakeholders for its appropriateness, feasibility and acceptability. Finally, the framework will be piloted to assess its effectiveness and suitability for full-scale implementation.

**Discussion:**

Currently, Ghana does not have a full-fledged HSPA tool that provides a holistic health sector-wide approach to assessing health system performance. Thus, developing this HSPA framework for the country will provide a tool for periodic and comprehensive assessment of the performance of the health system, which can be compared with that of other countries. Such a comparison will offer the opportunity for mutual learning and for exploring new options for formulating more effective national health policies. As this is expected to be the first attempt to develop a comprehensive HSPA framework in Ghana, this study will provide a basis for future discussions on how to further develop and implement HSPA programmes in the country.

## Background

Health systems are considered one of the largest sectors of the world’s economy and among the most important determinants of community development and social welfare [1]. In recent times, there has been an increased emphasis on improving the performance of health systems, especially in low- and middle-income countries (LMICs), to meet the health needs of the people. Policy-makers and development experts have realized that strong health systems are key to achieving and sustaining health gains [1, 2]. Increased attention on improving the performance of health systems has also been stimulated by the United Nations 2030 Agenda for Sustainable Development that calls for attaining health-related targets, including improving maternal health, reducing child mortality, achieving universal health coverage (UHC), and preventing and controlling a number of diseases that have a greater bearing on population health by the year 2030 [3]. There is now growing consensus that without strong health systems, achieving and sustaining the health-related components of the Sustainable Development Goals (SDGs) will be difficult if not impossible [4].

Assessing the performance of health systems through quantitative and qualitative methods is recognized as an effective approach to strengthening national health systems [5]. Health system performance assessment (HSPA) is a tool for gathering information about the functioning of a health system to inform policy decisions, monitor progress towards improved health, and identify best practices. Measuring the performance of a health system is considered an essential component in creating systems that are resilient, responsive, efficient, equitable, patient-focused, accessible and sustainable [5]. Further, HSPA is viewed as a step towards promoting transparency and accountability in healthcare services delivery. As a result, HSPA has now emerged as one of the priority areas of health systems research [6].

Measuring and improving the performance of a health system is not new. For instance, in the 1860s, Florence Nightingale pioneered systematic collection, analysis and dissemination of hospital data to understand and improve hospital performance [7]. The work on benchmarking by the Organisation for Economic Co-operation and Development (OECD), which since the mid-1980s has published a series of international comparative studies focusing on inputs into healthcare such as healthcare expenditure and human resources [8, 9], is another example. However, the first attempt to systematically measure the performance of health systems in a rigorous manner was based on the work of WHO, through its publication of the *World Health Report 2000*, where the performance of health systems in WHO Member States was comprehensively assessed [10, 11]. The latter in particular has stimulated wide-ranging debate about approaches to performance assessment both nationally and internationally [8].

Many high-income countries have since institutionalized HSPA as an integral component of their respective health systems [6, 12, 13]. However, few studies on HSPA have been documented in LMICs [14–17]. Examples of these few studies in LMICs include an analysis of district-level HSPA within the context of decentralization in Indonesia [18]; an assessment of the effect of health system reforms between 2001 and 2006 in Mexico, using a report card approach [19]; an evaluation of the performance of the healthcare delivery system in 16 states of India, using an econometric approach [20]; and monitoring of the rapid expansion of health services in Afghanistan, using a balanced scorecard approach [21]. In Africa, a few countries have institutionalized HSPA in their health systems. One of these is the Health Systems Trust of South Africa’s District Health Barometer, which monitors about 30 sets of indicators [22]. The Ugandan health ministry has also been producing an annual health system performance report since 2011, using league table analysis introduced in 2003 to compare performance among districts and determine “good” and “poor” performers, and the reasons why [15].

Over the past decade, Ghana has made continual advancements in using various assessment tools to monitor and evaluate the performance of the health sector. For instance, the Ghana Health Service (GHS) publishes “The Health Sector in Ghana Facts and Figures” that produces annual reports on the performance of some key health sector indicators. There is also the Maternal Health Survey jointly designed and conducted by Ghana Statistical Services (GSS) and GHS to provide data for monitoring key maternal health indicators including fertility levels, maternal mortality, family planning methods, pregnancy and postnatal care, abortion and miscarriage [22, 23]. Similarly, the GSS, in collaboration with other stakeholders in various sectors of government, researchers, civil society and international organizations, has implemented the Ghana Demographic and Health Survey (GDHS) programme that collects, analyses and disseminates information on demographic and health indicators such as housing and household characteristics, education, maternal and child health, nutrition, and knowledge and behaviour related to HIV/AIDS and other sexually transmitted infections (STIs) [24]. Furthermore, through the National Health Accounts (NHA), the country systematically and comprehensively monitors the flow of financial resources in the health system [25]. Moreover, the GHS, in collaboration with the University of Oslo, developed the District Health Information Management Software 2 (DHIMS2) in 2012 for reporting and analysing district health administration and health facility needs [26]. Data entered into DHIMS2 include indicators on finance, laboratory, pharmacy, disease control, maternal health, surgical operation and occupational health, among others [27]. Above all, the Ministry of Health (MOH) adopted a monitoring and evaluation framework called the Holistic Assessment Tool (HAT) during its 2007–2011 Programme of Work (POW) to monitor and assess progress towards achieving the objectives of the country’s Health Sector Medium Term Development Plans (HSMTDPs). It also serves as a feedback mechanism for development partners and other key stakeholders of the health sector. The framework has a set of indicators, milestones and targets clustered under the objectives of the national health strategy as defined in the HSMTDPs [28].

Despite the existence of the aforementioned assessment programmes, Ghana does not have a full-fledged HSPA tool that provides a holistic health sector-wide approach to assessing health system performance. For instance, our recent analysis revealed that the HAT is merely an assessment tool for monitoring and evaluating the MOH’s annual plans or POWs, as well as assessing progress towards the achievement of the country’s HSMTDPs [17]. According to WHO, a comprehensive HSPA is balanced in scope, and covers the whole health system and is not limited to specific programmes, objectives or levels of care. It is not bound by a reform agenda or national health plan end points [29 P141], as is the case with the HAT. Also, the HAT, in its current form, is not based on any specific conceptual or theoretical underpinning. As the literature indicates, a complete HSPA has three key components: a conceptual framework, an appropriate set of health system dimensions and a set of indicators measuring each of these dimensions [30]. The HAT has only health sector medium-term plan objectives (which keep changing any time a new medium-term plan is prepared) and indicators measuring the progress towards the attainment of these objectives. It is not clear whether the tool is based on the WHO model [10], the balanced scorecard system, the results-based model or the Donabedian model [31]. Further, the indicators under the HAT do not cover key health system dimensions such as the responsiveness of the health system and information systems for health [17].

The DHIMS2 platform, just like the HAT, has a number of limitations. For instance, it is more focused on health services delivery and not on the health system as a whole. Thus, it could be described as a sub-framework rather than as a health system framework. Also, since the health information management tool was developed by the GHS [26], other service delivery agencies feel reluctant to use the system for reporting [27]. For instance, some of the teaching hospitals in the country have a parallel electronic health records system, and this creates a challenge for the GHS in accessing their data [27]. As a result, the tool is not sufficiently comprehensive to provide a holistic assessment of the health system of the country.

Developing and implementing a Ghanaian HSPA tool that covers the entire health system will therefore bring the country in line with many developed and some developing nations in creating systems and frameworks that monitor and assess the performance of their health systems.

## Aims

**Fig. 1:**
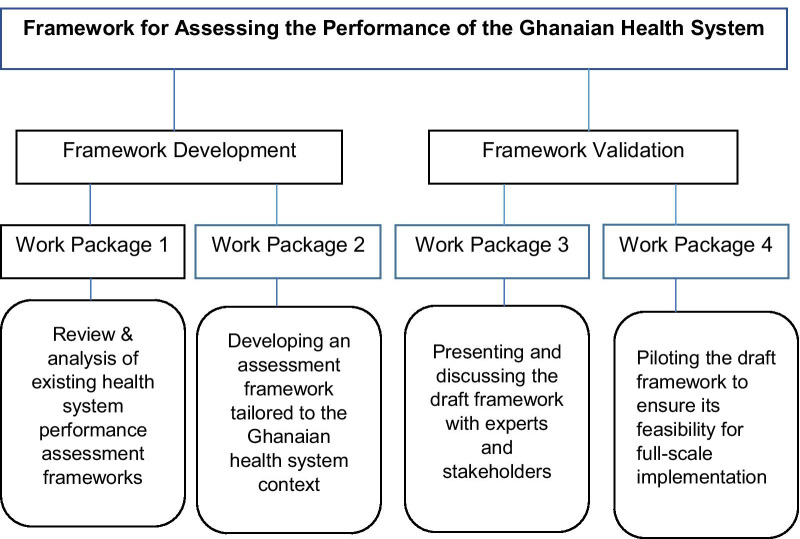
Overview of the study to develop a framework for assessing the performance of the Ghanaian health system

The overarching aim of this study is to provide a feasible/potential framework for periodic assessment of the performance of the entire health system in Ghana. This work will build on the previous efforts that have contributed to broader HSPA in the country, as indicated above. The study has four specific objectives classified into two main phases and linked to four work packages (Fig. 1).

Objective 1: To comprehensively review the literature and analyse the current national and international HSPA frameworks as tools for policy decision-making towards improved health.

Objective 2: Based on objective 1, to develop an assessment framework tailored to the Ghanaian health system context.

Objective 3: To present and discuss the draft framework with experts and stakeholders for its appropriateness, feasibility and acceptability.

Objective 4: To pilot the framework to ensure its effectiveness and suitability for full-scale implementation.

The research question is as follows: “Using the existing HSPA frameworks, how can an all-encompassing framework be designed for systematic monitoring and evaluation of the performance of the Ghanaian health system to ensure external accountability and internal quality improvement?”

## Methods

**Fig. 2:**
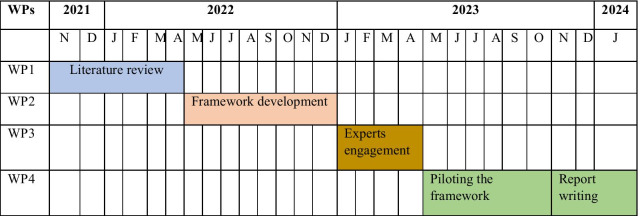
Timeline for developing a framework for assessing the performance of the Ghanaian health system

The study has four work packages (WPs): a literature review, development of an HSPA framework suitable for the Ghanaian context, presentation and discussion of the framework with experts and stakeholders, and piloting the framework. We will draw from a wide range of disciplines, including public health, health systems research, political science, health economics, health management information systems and quality management in healthcare. The study will start in November 2022 and will run until January 2024 (Fig. 2).

## Systematic review (WP1)

We will conduct a structured review to identify and examine the existing HSPA frameworks, especially the more established ones. The purpose of this task is to determine which framework or aspects of particular frameworks could be applied to the Ghanaian context. The review will inform the next stage of the study, where a draft framework will be developed with the input of the major important and relevant stakeholders, both public and private, in the health sector of Ghana and appropriate indicators extracted and mapped onto it.

A search strategy will be developed for key databases, including MEDLINE, CINAHL, PsycINFO, PubMed, Embase, Scopus, Science Direct and Google Scholar. We will include any type of report or peer-reviewed journal article that reports on HSPA frameworks. Our search will be limited to English-language publications between January 1990 (the year after which the vision of using large-scale data sources to help improve the performance of the healthcare delivery system as a whole became a reality) and November 2021. In addition to the database search, we will check the bibliographies of papers that will meet our inclusion criteria and contact the authors of identified frameworks to ask for any unpublished reports that will be considered relevant. Moreover, the websites of governmental entities and international organizations such as the OECD, European Observatory on Health Systems and Policies, the United States Agency for International Development (USAID), the World Bank, WHO, and Health Systems and Policy Monitor will be searched for relevant documents.

Frameworks with well-defined conceptual models and clearly stated dimensions will be included in the analysis. Each of the selected frameworks will be examined for congruence with the Ghanaian context, considering its conceptual outline and content, especially its dimensions. The dimensions most relevant to the Ghanaian health system will be discussed in detail and selected for inclusion in drafting the framework. Indicators contained in each dimension will also be analysed for inclusion in our indicator selection.

## Development of Ghana’s HSPA framework (WP2)

Following the literature review, a draft performance assessment framework will be developed in partnership and collaboration with the major stakeholders in the health sector for the Ghanaian health system. In this regard, we will work in relationships with the following important stakeholders over the duration of the project: the MOH, the GHS, the Christian Health Association of Ghana, the teaching hospitals, the private health service providers, the Ghana Medical Association, the Registered Nurses and Midwives Association, the health sector development partners and public health experts, among others. This will ensure that they are part of the codesign and framework development process from the onset. In this way, sufficient accounting of the multifunctional complexity of healthcare delivery in a health system—requiring trade-offs, for example, between prevention and treatment or between primary and specialized care [8], which are major challenges in developing health systems assessment tools—will be adequately addressed.

Two main tasks will be performed at this stage: drafting a conceptual framework with an appropriate theoretical underpinning and clearly defined health system dimensions based on the input of all these important stakeholders; and selecting performance indicators for each of the dimensions of the draft framework.

### Drafting a conceptual framework

The frameworks analysed and selected during the literature review will be grouped into various conceptual themes, such as goal-driven framework, the Donabedian structure–process–outcome model [28], quality-based framework, results-based logic model and balance scorecard system. With the important stakeholders, we will then discuss and agree on a more harmonized and integrated conceptual framework or parts of particular frameworks suitable for adoption for Ghana. To guide our discussion, we will define the main objectives of the HSPA within the broad context of the national health system goals, priorities and strategies. Having adopted a framework for Ghana, the next step will be selecting appropriate health system dimensions that are applicable to Ghana’s health system, taking into consideration the local and cultural context. This is critical, as cultural competence or the lack thereof has been identified as a key factor affecting performance in most health systems [32]. All dimensions within the selected HSPA frameworks during the literature review will be listed and individually discussed for inclusion or exclusion for Ghana’s HSPA framework.

### Selecting and mapping indicators to the draft framework

A broad range of indicators covering major aspects of the Ghanaian health system will be selected and mapped to the dimensions of the draft framework. Our aim is to select indicators that are both relevant to the local context and externally comparable. Thus, our indicator selection will come from two main sources: drawing from internationally based indicators and selecting from a list of local indicators that are collected routinely through the implementation of various health strategies, policies and assessment programmes. The internationally based indicators will be mainly those identified through the literature search. With the locally based indicators, we will first list all the existing health policies, strategies and assessment programmes. This will be followed by detailed scrutiny of each of these strategies, policies or assessment programmes for the appropriate and relevant indicators to be extracted.

A set of three selection criteria will be used as guiding principles to assess each of the extracted indicators for inclusion in the draft framework. These are (1) the relevance of the assessment indicator to the health system in terms of its ability to provide useful information for policy-makers to take specific actions to improve service delivery and health outcomes; (2) the feasibility of obtaining data for the measure; and (3) the scientific soundness of the indicator in terms of validity, reliability and accuracy. Each indicator will be internally scored on a scale of 1–5 on these criteria by two members of the research team, key stakeholders and identified health systems experts working independently. This is to add rigour and robustness to the scoring system, thereby strengthening the validity, reliability and accuracy of the indicators and overall framework. The cumulative results will be compared to generate a list of indicators that will score more than 50% of the median score. The internal assessment will enable us to reduce the number of the extracted indicators to a more manageable number, which will then be subjected to further external scoring and validation in the next stage of the project.

## Presenting and discussing the draft framework with experts and stakeholders (WP3)

After selecting and mapping appropriate indicators to each of the dimensions of the draft HSPA framework, we will present the tool for external validation. This will involve two main stages. First, we will organize a workshop for a detailed discussion on the appropriateness of the framework with respect to the needs and circumstances of the Ghanaian health system. Participants will be drawn from academia (health-related academics), the MOH (mainly senior civil servants), GHS, health sector development partners, and managers and senior clinical leaders within the Ghanaian healthcare delivery system (both public and private). The framework will be presented and explained to the participants, after which a discussion will follow. Feedback from the participants will be used to revise the draft framework.

The next stage will involve an expert panel discussion, where the shortlisted indicators will be presented for external scoring. We will develop a list of health policy and systems research experts with extensive publication and professional experience in the fields of health systems reform and strengthening, health planning and management, global health, health economics, health promotion, human resources for health, health quality management, epidemiology (communicable and noncommunicable diseases), and health management information systems within the context of developing countries. Participants will be asked to score each of the shortlisted indicators according to the same set of criteria that will be used for the internal assessment, as described earlier. The panel members will be allowed to discuss and resolve any ambiguity related to selection and content of the indicators. A mean score will be computed for each indicator by summing all ratings reported for a single item. Subsequently, the indicators will be listed in descending order of priority, and with the consensus of all members in the expert panel, the final set of indicators will be selected for inclusion in the framework development.

## Piloting the draft framework (WP4)

The final stage of our work will involve testing the suitability of the draft framework by using it to assess the performance of the Ghanaian health system. The piloting, which will be in the form of a feasibility study, will help us determine data availability and data generation for all of the selected indicators, as well as ensuring sound and meaningful interpretation of reports that will be generated from the assessment programme.

We will collect and analyse data pertaining to the final set of indicators included in the framework. Requests will be made to the MOH, GSS, GHS and all other ministries, departments and agencies that are involved in healthcare provision in the country, to request data between 2010 and 2020. The performance of each indicator, in terms of trend over time and international comparison, will be deduced from the collected data. International comparison will be mainly carried out with the WHO African Region. Points ranging from 0 to 2 will be allotted for the two bases of comparison as shown in Table 1. The sum of each category will be calculated for each indicator to derive the overall assessment score, ranging from very good (4) to very poor (0).

**Table 1:** How each indicator will be classified and assessed

No.	Indicator	Trend over time	International comparison	Assessment
		Improving = 2	Ghana fares better = 2	Very good = 4
		Stable = 1	Ghana fares same = 1	Good = 3
		Deteriorating = 0	Ghana fares worse = 0	Satisfactory = 2
				Poor = 1
				Very poor = 0

Once the assessment scores for the indicators are calculated, we will compute the overall score for each dimension using the sum of the scores of each indicator within each dimension. A classification similar to that of the indicators—that is, from very good (4) to very poor (0)—will be produced for each dimension.

Having completed the analysis, we will put together an assessment report and again invite health policy and systems research experts to review the assessment methodology, especially the scoring and classification systems for the indicators and their respective dimensions, as well as interpretation of the results. The framework will be amended to incorporate suggestions and/or recommendations from the experts. Also, indicators with no readily available data for their measurement will be excluded from the final draft framework. We expect to conclude this project with a formal HSPA framework with clear and unambiguous dimensions that are linked to the values and priorities of the Ghanaian health system.

## Discussion

We strongly support the argument that developing a robust conceptual framework within which specific performance measures could be tested and implemented regularly is a major requirement for a performance measurement system in any country [5]. The framework we intend to develop will cover all of the major dimensions of the Ghanaian health system. We will ensure that the framework aligns with the health system objectives of Ghana, is integrated with information technology (IT) and routine data collection in the country, includes high-priority and hard-to-measure areas, and has measurement indicators that are internationally comparable.

A major benefit of developing this HSPA framework for Ghana is that it will provide a tool for periodic and comprehensive assessment of the performance of the health system, which can be compared with that of other countries. Such a comparison will offer the opportunity for mutual learning and for exploring new options for formulating more effective national health policies. The framework is also expected to be added to the existing assessment tools that are being used to measure progress towards attaining UHC and the health-related components of the SDGs. Furthermore, as this is expected to be the first attempt to develop a comprehensive HSPA framework in Ghana, our work will provide a basis for future discussions on how to further develop and implement HSPA programmes in the country.

There is no doubt that including a large set of indicators will improve the content validity of each dimension of the framework. However, to avoid collecting and presenting an overwhelming number of indicators, which could result in unreasonable burden of data collection and analysis, we will ensure that the number of indicators of the framework stays within acceptable limits. We will be guided by the experience of other countries in developing their HSPA frameworks and will limit the number of indicators to a manageable number so that trends in performance can be effectively monitored.

One major limitation we anticipate is a situation of gaps in data availability to measure some of the important indicators we will identify. This might result in the exclusion of key assessment indicators from the HSPA framework. Also, because we will use existing HSPA frameworks in developing our framework, and few performance assessment frameworks have been developed in low- and middle-income countries, there is a greater challenge in the need to avoid conceptualizing a framework that is more apt for a high-income country setting, which may essentially differ from situations prevailing in Ghana.

We intend to present and explain the final work to the authorities of the MOH to increase the chances of the Ministry’s adoption of the framework for formal assessment of the performance of the health system at the national level. Also, academic dissemination will be done through publication of the output of the project in a per-reviewed, open-access healthcare journal and presentations at conferences.

## Data Availability

The data used in this analysis are available from the corresponding author on request.

## Abbreviations

HSMTDP: Health Sector Medium Term Development Plan
HSPA: Health system performance assessment
MOH: Ministry of Health
